# Neural Correlates of Developmental Speech and Language Disorders: Evidence from Neuroimaging

**DOI:** 10.1007/s40474-014-0019-1

**Published:** 2014-06-07

**Authors:** Frédérique Liégeois, Angela Mayes, Angela Morgan

**Affiliations:** 1 UCL Institute of Child Health, Cognitive Neuroscience and Neuropsychiatry Section, 30 Guilford Street, London, WC1N 1EH UK; 2 Language & Literacy Group, Murdoch Childrens Research Institute, Flemington Road, Parkville, Victoria Australia; 3 Department of Paediatrics, University of Melbourne, Parkville, Victoria Australia

**Keywords:** Speech disorder, Language disorder, Specific language impairment, Speech delay, Speech sound errors, Childhood apraxia of speech, Motor speech disorder, MRI, Functional MRI, Diffusion-weighted MRI, Communications disorders, Childhood

## Abstract

Disorders of speech and language arise out of a complex interaction of genetic, environmental, and neural factors. Little is understood about the neural bases of these disorders. Here we systematically reviewed neuroimaging findings in Speech disorders (SD) and Language disorders (LD) over the last five years (2008–2013; 10 articles). In participants with SD, structural and functional anomalies in the left supramarginal gyrus suggest a possible deficit in sensory feedback or integration. In LD, cortical and subcortical anomalies were reported in a widespread language network, with little consistency across studies except in the superior temporal gyri. In summary, both functional and structural anomalies are associated with LD and SD, including greater activity and volumes relative to controls. The variability in neuroimaging approach and heterogeneity within and across participant samples restricts our full understanding of the neurobiology of these conditions— reducing the potential for devising novel interventions targeted at the underlying pathology.

**Electronic supplementary material:**

The online version of this article (doi:10.1007/s40474-014-0019-1) contains supplementary material, which is available to authorized users.

## Introduction

Developmental communication disorders are prevalent, affecting over 10 % of school aged children [1]. Here we focus on two common subtypes, namely Language (LD) and Speech (SD) disorders. Whilst some symptoms may “resolve” or be compensated for into adolescence [2], there is increasing evidence for persistent life-long negative impacts of SD and LD on literacy, educational, employment, and psychosocial outcomes [3–5, 6•, 7]. Traditionally, both LD and SD have been defined as idiopathic (of unknown origin). Clearly the term idiopathic implies that the disorders cannot be explained by neurological or sensory deficits, nor are they associated with frank brain abnormalities on clinical MRI. Advances in neuroimaging methods over past decades however, have uncovered both functional and sub-macroscopic structural brain anomalies associated with these disorders.

Language Disorders (LDs) are defined as a failure to develop age appropriate language skills despite normal sensory abilities and environmental exposure, and affect between 7 % and 20 % of pre-schoolers [8, 9]. A spectrum of LD profiles exists, dependent upon which aspect of language processing is most impaired (e.g., syntax, semantics) [10]. LDs have in the past also been termed “Specific Language Impairments” or SLI, but the “specific” aspect of the disorder remains controversial [11•]. Speech Disorders (SDs) is also an umbrella term, encompassing numerous subtypes of developmental speech disorder. Several classification methods have been proposed for SDs [12••, 13]. Here we consider studies that focus on subtypes of articulation disorder (phonetic based or motor execution errors), phonological disorder (phonemic based or cognitive-linguistic errors), and childhood apraxia of speech (CAS, motor planning and programming errors), as well as those that use the less explicit diagnostic terms of speech errors and speech delay. Although behavioral assessments of deficits are crucial, neuroimaging studies can provide us with a different level of explanation of symptoms, and may offer a novel way of classifying subtypes of SDs and LDs.

To date, the most extensive neuroimaging studies of a developmental speech and language disorder have been carried out in the affected members of the KE family, who have a rare mutation in the FOXP2 gene, with a seminal imaging study published on this family in 1998 [14]. Affected members of the KE family present with both speech (verbal and orofacial praxis and dysarthria) and language impairments, affecting speech intelligibility as well as the use of morphosyntax and the comprehension of complex grammatical structures [15, 16]. It is critical to note that the phenotypic marker, co-segregating affected and unaffected family members, is a diagnosis of CAS. Since the early KE studies, examination of the neural basis of SD and LD has been limited and is still an emerging field.

Here we systematically reviewed all articles published between 2008 and 2013 in individuals (adults or children) diagnosed with developmental forms of SD or LD.^1^ We present functional and structural MRI findings to ask whether we are any closer to answering the following question: which brain anomalies are associated with atypical development of speech and language?

## Methods

### Search Strategy

A computerized systematic search was conducted of relevant databases: EMBASE (1996 to August 2013), OVID MEDLINE (1996 to August 2013), PubMed (searched August 2013). The following MeSH terms were used to identify SD and LD papers of interest: (speech disorder OR articulation disorder OR phonetic disorder OR speech delay OR phonological impairment OR language disorder OR language development disorder) AND (magnetic resonance imaging OR diffusion magnetic resonance imaging OR echo planer imaging OR computerized positron emission tomography OR single photon OR brain) NOT (dyslexia OR Asperger syndrome OR autistic disorder OR aphasia OR Broca’s aphasia OR Wernicke’s aphasia OR primary progressive aphasia OR conduction primary progressive non fluent aphasia OR electroencephalography). Of note, the MeSH terms for SDs and LDs were kept broad to encompass all relevant terminology (e.g., speech delay, speech sound disorder, SLI). Searches were limited to papers written in English between 2008 and present (August 2013) with human participants. Manual searches were completed in relevant journals publishing brain-behavior relationships in this field (i.e., Brain and Language, Brain Topography).

### Inclusion Criteria

Studies were included if they reported results of individuals with either SD or LD, together with a MRI neuroimaging method to investigate brain structure or function. Full text articles were required to be available and published in English. Failure to meet one of the above criteria resulted in exclusion.

### Data Extraction

A total of 2,602 abstracts were identified. An additional four were located in a manual search. Two stages of exclusion were conducted (Supplementary Fig. 1). Firstly, papers were excluded based on title only, including any duplicates (n = 2,573) by one author (A. Mayes). Secondly, papers were excluded based on independent review of the abstract and/or full text article (n = 23) by all three authors, using the following criteria: Participant selection criteria (excluding studies with children who have brain injury); imaging methods (excluding studies without imaging); analysis method (excluding studies with no quantitative analysis). Disagreements were resolved by discussion (one article). All three authors manually searched for additional publications relevant to the field published between 2008 and 2013 and listed within the reference list of each selected paper.

### Critical Appraisal

To examine the level of evidence provided, we used the NHMRC (National Health and Medical Research Council, Australia) classification (http://sydney.edu.au/medicine/21st-century/presentations/2013/NHMRC-hierarchy-of-evidence.pdfref/Appendix) [17]. This classification system allows a grading from the poorest level of evidence (Grade IV, Cases series studies) to the highest (e.g., Grade I, systematic review of randomized controlled trials).

## Results

### Overview of Articles: Methodological Considerations and Critical appraisal

Ten articles (see Supplementary Fig. 1) were included, five on SD and five on LD. All were case-control studies (NHMRC evidence level III-2) [17]. Effect sizes for group comparisons were available for two studies (Preston et al., [18], Table 1; Lee et al., [19], Table 2) and could not be calculated for the remainder as standard deviations were not provided. Age bands were relatively narrow (1–3 years) for studies on children with SD, but wider in studies on children with LD, where three out of four studies reported on groups spanning nine years or greater. Only Verhoeven et al., [20] focused on a narrow age band (all cases were 10 year olds).

**Table 1:** Neuroimaging studies on SD

Article	Sample characteristics (study group)	Sample size (males)	Mean age (range)	Methods	Brain behaviour correlations	Decreases in study group (effect size)	Increases in study group (effect size)
Preston et al., 2014	Speech sound errors (SSE) Persistent errors Phonetic analysis <70 % PPC on ≥1 sound [79] No difference on CTOPP [80] between TD and SSE groups	SSE (n = 23, 18 M) TD (n = 54, 30 M)	SSE: 9y 9 m TD: 9y 11 m Total: 8y 6 m to 11y 11 m	VBM (whole brain)	No significant correlations between speech sound accuracy and gray and white matter in the SSE group alone	Reduced grey matter: R lingual gyrus ( d = 0.86) Reduced white matter: R lateral occipital gyrus (d = 0.95)	Increased grey matter: L Heschl's gyrus, L planum temporale, inferior L SMG LSTG (d = 1.05), R planum polare, R Heschl's Gyrus, R planum temporale (d = 0.95). Increased white matter : Splenium and anterior CC extending to cingulate (d = 0.83)
Kadis et al, 2013*	Childhood apraxia of speech (CAS) Moderate to severe , no dysarthria* Phonetic (GFTA)^a^[81] and phonemic (HCAPP) analysis^a^[82] VMPAC (focal oromotor; [83] Sequencing)^a^	CAS (n = 11, 8 M) TD (n = 11, 5 M)	CAS: 4.7y TD: 4.8y	Cortical thickness ROIs in both hemispheres: IFG-PO; posterior SMG; posterior STG; inferior pre- and post-central gyri	No correlation between L SMG & any speech performance measures	None	Increased cortical thickness: L SMG
Liegeois et al, 2011	Affected members of KE family (FOXP2 mutation) CAS with mixed dysarthria	KE: (n = 4, 2 M) TD: (n = 4, 2 M)	Adults (not specified)	FMRI Overt nonword repetition VS. Listening to white noise ROIs: putamen		Reduced brain activity:L cerebellum (lob IX), R anterior cingulate, L + R MOG, R SFG, R SMA, L lingual gyrus, L rolandic operculum (extending into precentral gyrus), L + R putamen (small volume correction);	Increased brain activity Not reported
Tkach et al., 2011	Speech sound disorder (SSD) – History moderate to severe SSD (GFTA & KLPA). [81, 84] 5/6 typical adult level production by school age and time of scan Phonetic & phonemic analysis	SSD: (n = 6, 5 M) TD: N = 7**	Adolescents: SSD: 17y TD: 18y	fMRI Overt nonword repetition VS. Rest		Reduced brain activity: R IFG (BA45 + BA46); R MTG	Increased brain activity: L postcentral gyrus; L SFG; L + R MFG; Lmedial frontal gyrus; L IFG (BA47); L sub-gyral frontal lobe; L STG; L AG; L IPL; L SMG; L CG; L + R IOG; R cuneus; R lingual G; L + R MOG; L putamen; L hypthothalamus L + R declive R culmen.
Preston et al., 2012	Speech sound errors (SSE) Persistent errors Phonetic analysis <70 % PPC on ≥1 sound [79] Large effect sizes for group comparison (TD vs SSE) on some WJ, [85] CTOPP [80] subtests	SSE: (n = 17, 14 M) TD: (n = 17, 14 M)	SSE: 9y 7 m TD: 9y 10 m Total Range: 8y 6 m to 10y 10 m	fMRI Auditory and visual presentation, word and nonwords. Tasks: Covert naming, matching using button press		Reduced brain activity: Auditory presentation: L + R orbital gyrus; L temporal pole; R ITG; L MTG Visual presentation: L SOG, L cerebellum	Increased brain activity: Auditory presentation: L + R inferior SPL; precuneus; R SMG and Postcentral gyrus; L fusiform; L + R STG; posterior & anterior cingulate; R MFG; cuneus; L gobus pallidus; R lingual G; L cerebellum; R temporal pole; L MFG; R SFG; L middle occipital gyrus; L insula; R precentral gyrus Visual presentation: precuneus, R STG/STS, anterior cingulate, L + R fusiform, posterior cingulate, L MFG/SFG, L STS/MTG, L SPL, R MTG, L globus pallidus, R anterior IPS, R lingual gyrus, L postcentral gyrus, L IFG, R precentral gyrus, L + R MFG, R parahippocampal gyrus

Study Group refers to SSD, SSE or CAS here. Note that diagnoses were made by the authors

*Abbreviations: AG* angular gyrus, *ASD* Autistic Spectrum Disorder*, CAS* childhood apraxia of speech*, CC* corpus callosum, *CG* cingulate gyrus, *CTOPP* Comprehensive Test of Phonological Processing, *GFTA* Goldman Fristoe Test of Articulation, *CAPP* Hodson Computerised Analysis of Phonological Processes, *IFG* inferior frontal gyrus, *IOG* inferior occipital gyrus, *ITG* inferior temporal gyrus, *KLPA* Khan-Lewis Phonological Analysis, *L* left hemisphere, *LI* language impairment, *MFG* middle frontal gyrus, *MOG* middle occipital gyrus, *MTG* middle temporal gyrus, *PPC* percentage of consonants correct*, r* right hemisphere, *ROI* region of interest, *SFG* superior frontal gyrus, *SMA* supplementary motor area, *SMG* supramarginal gyrus, *SOG* superior occipital gyrus, *SPL* superior parietal lobule, *SSD* speech sound disorder, *SSE* speech sound errors, *STG* superior temporal gryus, *STS* superior temporal sulcus, *TD* typical development, *VBM* voxel based morphometry, *WJ* Woodcock-Johnson

* Note that this is an intervention study and we will here only review neuroimaging findings reported before intervention—where the SSE and control groups are of equal size.

** Gender not reported

^a^Only report magnitude of difference after therapy

**Table 2:** Neuroimaging studies on LD

Article	Study group and selection criteria	Sample size (males)	Mean age (range) in years	Methods	Brain behaviour correlation	Decreases in study group (effect size)	Increases in study group (effect size)
Badcock et al 2012 [29]	SLI <10th percentile on ≤ 2 language or literacy tests (Ax: CCC-2 [59] or CC-A [60], TROG-2 [61], TOWRE [62], NEPSY [63], and ≥ 80 WASI [64].	SLI (n = 10; 9 M); SIB (n = 6; 4 M); TD(n = 16; 7 M)	1. SLI: 13.5 (8–17) 2. SIB: 18 (12–22), 3. TD: 12.50 (6–25)	VBM (whole brain) fMRI Silent word association task (“Speech”) Vs. Passive listening to reversed speech	Not examined	Reduced grey matter: SLI < TD: Medial frontal pole, L + R pSTS ext. to R STG, R medial superior parietal cortex, L occipital pole, R caudate nucleus, R substantia nigra; R pMTG SLI < SIB: L + R par operculum cortex, L occipital pole Reduced brain activity Speech condition: SLI < TD: L IFG (pars orbitalis ); SLI < SIB: L IFG (pars orbitalis); R IFG (pars triangularis); L pSTG; Speech > Reversed Speech: SLI < TD: L pSTG; R putamen; SLI < SIB: L IFG (pars orbitalis)	Increased grey matter: SLI > TD: L frontal operculum, R anterior insula, L aIPS; SLI > SIB: L aIPS Increased brain activity None
Verhoeven et al, 2012 [20]	SLI (mixed receptive-expressive) <3rd centile ≥1 of 3 subtests of Reynell Taaloontwik-kelingsschalen [65], Taaltests voor Kinderen [66], or Schlichting Test voor Taalproductie [67], and PIQ or FS IQ > 80 [68]	SLI (n = 13; 10 M) TD (n = 12; 8 M)	SLI: 10.1(SD = 0.4) TD for SLI: 10.2(SD = 0.3);	DTI Tractography (Superior Longitudinal Fasciculus, SLF)	In SLI group: WCR subtest and FA both L and R SLF; WCE and left SLF	SLI < all TD: Reduced Fractional anisotropy in SLF	No other ROI measured
de Guibert et al, 2011 [27]	SLI >1SD below mean for phonology, sentence repetition, and morphosyntactic integration [69, 70], and WISC/WAIS [71, 72] ≥ 70	SLI (n = 21, 9 M) TD (n = 18, 9 M)	SLI: 11.4 (7–18) TD: 12.7 (8.7–17.7)	fMRI Silent generation or naming. ROI analyses		Reduced brain activity: Auditory Response Naming: L pSTG/SMG junction	Increased brain activity Phonological difference task: R anterior insula ext to IFG opercularis/ triangularis and caudate head.
Soriano-Mas et al 2009 [25]	DLI Rapin [26] classification: Speech programming deficit (n = 5), phonological-syntactic deficit (n = 18), lexical deficit (n = 8), mixed (n = 5) >1SD below mean PPVT [73], TTFC [74], ITPA [75], and WISC-III [76]. IQ > 85.	SLI (n = 36; 24 M) TD (n = 36; 24 M)	SLI: 10.58(5–17) TD: 10.88 (5–17)	VBM	Older SLI : negative correlation btw verbal IQ and GM R perisylvian region, PPVT + GM occipital petalia	None	Increased grey matter Global volume; R posterior perisylvian, L MOG (occipital petalia) Young SLI > Young TD: L + R entorhinal, L + R temporopolar, L + R caudate nucleus, L + R precentral gyrus, L precuneus, L medial MOG Increased white matter Global volume; Young SLI > Young TD: R medial front cortex, L + R MTG
Lee et al.,2013 [19]	Developmental Language Impairment (DLI) >1.5 SD below mean of language composite (word derivations – subtest of TOAL-4, PPVT-4, token test) [77, 78]. WASI PIQ assessed not used as criteria	DLI (n = 12; 4 M) TD (n = 12;4 M)	DLI: 21.99 TD: 22.06 Overall range 19–25	DTI + Volumetric ROIs: Caudate nucleus; putamen; nucleus accumbens; globus pallidus; thalamus; occipital, parietal, temporal, frontal lobes; hippocampus	negative correlation btw nucleus accumbens; globus palladius; putamen, hippocampus ROIs, whole brain FA + language composite	Reduced grey matter volumes: ICV, L + R caudate nucleus (d = -1.21), L + R thalamus (d = -1.57), occipital lobe (d = -1.54), parietal lobe (d = -1.47), temporal lobe (d = -1.32), frontal lobe (d = -1.50). Reduced FA: Whole brain (d = -2.00); globus pallidus (d = -0.96) thalamus (d = -1.41), occipital lobe (d = -2.25); parietal (d = -1.74); temp (d = -1.58) front (d = -2.27)	Increased grey matter Volumes: When ROI volume corrected for ICV: putamen (d = 1.07), nucleus accumbens (d = 1.0); hippocampus (d = 1.70). Increased FA: None

Study group here refers to SLI or DLI. Note that diagnoses were made by the authors themselves

*Abbreviations: aIPS* anterior inferior parietal sulcus, *CC-2* Children’s communication checklist version 2, *CC-A* Communication checklist for adults, *DLI* developmental language impairment*, DTI* diffusion tensor imaging, *FA* fractional anisotropy, *GM* grey matter, *ICV* intracranial volume, *IFG* inferior frontal gyrus, *ITPA* Illinois test of psycholinguistic abilities, *MOG* middle occipital gyrus, *MTG* middle temporal gyrus, *NEPSY* NEuroPSYchology, *PIQ* performance intelligence quotient, *pMTG* posterior middle temporal gyrus*, PPVT* Peabody picture vocabulary test, *pSTG* posterior superior temporal gyrus, *pSTS* posterior superior temporal sulcus, *ROI* region of interest, *SFL* superior longitudinal fasciculus, *SIB* sibling, *SLI* specific language impairment, *STG* superior temporal gyrus, *TD* typically developing, *TOWRE* Test of Word Reading Efficiency, *TROG-2* Test for Reception of Grammar-2, *TTFC* token test for children, *VBM* voxel based morphometry, *WAIS* Wechsler Adult Intelligence Scales, *WASI* Wechsler Abbreviated Scales of Intelligence, *WCE* word classes expressive (CELF subtest), *WCR* word classes receptive(CELF subtest), *WISC* Wechsler Intelligence Scales for Children.

Another observation is that the recruitment samples in both SD and LD studies were heterogeneous with regard to diagnosis. Liegeois et al., [21] and Kadis et al., [22] focused on CAS, although the former was focused on FOXP2 associated CAS in adults and the latter included young idiopathic cases.

Tkach et al., [23] considered both phonetic (articulatory) and phonemic (phonological process analysis) level errors. Preston et al., [18, 24] report on phonetic level errors only, using largely the same sample of children with persistent SD (17 of the 23 in 2014 were from the original study [24]). Phonological process analysis was not reported in either Preston et al., study, however CTOPP results were reported, i.e., a measure of phonological awareness, rather than production per se. No group differences were reported on the CTOPP as a whole in Preston et al., [24], but moderate effect sizes were reported on CTOPP subtests of Elision and Blending words in the later study [18]. Hence, it is challenging to interpret the level of phonological deficit, if any, in these participants who are denoted as having “Speech Sound Errors” (SSE). All studies included participants with persistent SD, with the exception of Tkach et al., [23], who focused on a sample with a *history* of SD. Only one of the six cases in Tkach et al., had persistent SD.

Thus, overall, across the five SSD studies, it appears that one focused on persistent speech motor programming deficits (CAS) [22]; one on persistent speech programming and execution deficits associated with FOXP2 mutation (CAS and dysarthria) [21]; two on persistent phonetic level (i.e., articulatory) deficits [18, 24]; and one on a history of phonetic and/or phonemic level (i.e., articulatory/phonological) deficits [23].

Similarly, for the LD studies, inclusion criteria and diagnoses were highly varied. Some even included several subtypes of impairment within LD groups. Verhoeven et al., [20], included children with a history of language delay and who scored <10^th^ percentile on at least one of three language tests beyond the age of four. At the time of testing, the SLI group scored more than one standard deviation below the normative mean on both receptive and expressive subtests of the Dutch CELF. The study by Soriano-Mas et al., [25] included children with speech programming, phonological-syntactic, lexical, or mixed deficits according to the Rapin criteria [26]. At the time of testing, the SLI group scored more than one standard deviation below the normative population mean on three language measures. The notable exception is the study by de Guibert et al., [27], which claims to focus exclusively on young people with “structural” language impairment. Participants with LD showed deficits in phonology (assessed using unfamiliar word repetition, which is arguably not a pure test of phonological ability), morphosyntax (tested using a sentence completion test), or both. Finally, the study on adults [19] included participants diagnosed with LI as children, and who as an adult group scored 1.5 standard deviation below the normative mean on a composite language score. It is noteworthy that the classification of LD is still a matter of debate, with the question of a continuum vs. discrete entities still unanswered [11•, 28].

### Neuroimaging Findings in SD (Table 1)

#### Structural imaging (Fig. 1b)

Two structural imaging articles on SD met inclusion criteria; one investigating cortical thickness measurements in children diagnosed with CAS [22], and the other using a whole brain approach (Voxel Brain Morphometry-VBM) in children with speech sound errors (SSE) [18]. Interestingly, both studies reported on greater grey matter within the left supramarginal gyrus (SMG) for the groups with SD. Unlike Kadis et al., [22], Preston et al., [18] additionally reported increases in the right SMG and bilaterally in the planum temporale and Heschl gyrus in children with SSE.

**Fig. 1:**
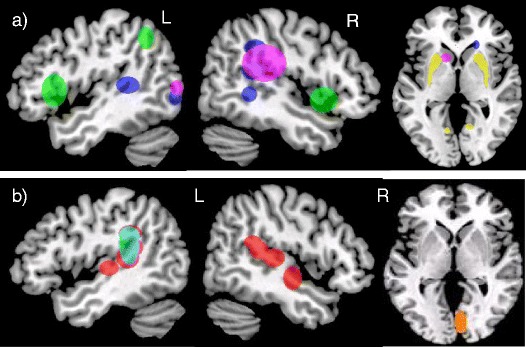
Morphological grey matter (*GM*) differences in individuals with (**a**), Language Disorder (*LD*) and (**b**), Speech Disorder (*SD*), relative to typically developing participants *Colour code*: GM volume decreases in LD: Badcock et al., [29] = *blue*; GM increases in LD: Badcock et al., [29] = *green*; Lee et al. [19], relative volumes) = *yellow*; Soriano Mas et al., [25] = *purple*; GM increases in SD: Kadis et al. 2013 = *light blue*, Preston et al., [18] = *red*. GM decreases in SD: Preston et al., [18] = *orange*. Note: Fractional anisotropy (*FA*) differences (Lee et al.) are not illustrated here as changes were observed across the whole brain (Table 2)

### Functional Imaging

Two of the three functional MRI studies included examined adults with persistent SDs, and both used overt non-word repetition tasks. The first focused on the affected members of the KE family with persistent CAS [21], while the other [23] focused on adults with a history of SSD. Perhaps not surprisingly, results were inconsistent, one reporting left sided hypo-activation in a wide articulatory network (rolandic operculum, primary motor cortex, cerebellum, and putamen); the other reporting hypo-activation in the right hemisphere, namely the middle temporal and inferior frontal gyri (IFG, Brodmanns’ area 45–46). The study by Tkach et al., [23] also reported widespread hyper-activation mainly in the left hemisphere including in the putamen, IFG, SMG, and superior temporal gyri (STG).

The third study [24] used a range of fMRI tasks requiring participants to press a button to signal a match or mismatch between a picture cue and a subsequent stimulus. Stimuli were words or pseudo words and were presented in auditory or visual (print) modalities (see Table 1). A widespread network of regions was over or under-activated in the group with SSE, some located within the typical language network (i.e., overactive STG and SMG for auditory presentation); and others external to this network (e.g., underactive orbital gyri, overactive middle frontal gyrus and posterior cingulate for auditory presentation). Of note, participants with SSE showed more activation in the left inferior/middle frontal gyrus when presented with words rather than with pseudo-words, while the control group showed the opposite trend.

### Neuroimaging Findings in LD (Table 2)

#### Structural Imaging (Fig. 1a)

Of the four structural studies included [19, 20, 25, 29], all but one focused on child participants [19]. Two VBM studies reported abnormalities in LD participants within the temporal region, with some degree of anatomical inconsistency. One reported reduced grey matter in the right posterior superior and middle temporal gyri and left posterior superior temporal sulcus [29]. The other, on the contrary, reported increased regional volumes within a right posterior “perisylvian” area extending from the posterior STG to the angular and SMG [25].

Subcortical structures were also found to develop atypically in participants with LD, with again contrasting findings for the caudate nucleus (reductions in two papers [19, 29]; and increases in one [25]). Of note, reductions were also found in LD participants’ unaffected siblings [29]. The same study also found that caudate nucleus volume was negatively correlated with non-word repetition scores in children with LD [29]. Larger relative volumes (i.e., corrected for intracranial volume) were reported bilaterally in the putamen for children with LD in one study, with a larger putamen associated with poorer language performance [19].

Soriano-Mas et al., [25] examined white matter using VBM. They reported morphological increases in white matter bilaterally in the middle temporal gyrus and an anterior cluster in the medial frontal lobe for the younger SLI group. Two studies used diffusion-weighted MRI to examine microstructural abnormalities in LD. Verhoeven et al., [20] focused on the superior longitudinal fasciculus, and reported reduced fractional anisotropy (FA) values (a measure of white matter microstructure) for children with LD. Additionally, FA values were negatively correlated with language measures including word class receptive and expressive sub tests. In contrast, the study by Lee et al., [19] focused on grey matter. They reported volumetric reductions in most of the subcortical and cortical ROIs examined, and FA reductions in the cortex, but no FA reductions in the caudate or putamen. Poor language performance was only associated with FA reductions across the whole brain.

### Functional Imaging

Two fMRI studies of children with LD were included. One employed covert lexical semantic and phonological tasks [27], and the other a covert auditory response naming task [29]; and both reported hypo-activation of the posterior STG. In one study, right sided hyper-activation was seen within the right insula extending to IFG -*pars opercularis*/*pars triangularis*, and caudate head for children with LD in response to a phonological difference task (i.e., where the children see a picture and silently generate names of three objects, each with a different initial phoneme) [27].

## Discussion

All studies reported significant developmental anomalies of brain structure or function in relatively small groups of children with SD and LD as revealed by quantitative imaging. Here we discuss the most consistent findings, but emphasize the need for caution in interpretation given methodological variability across studies.

### Neural Basis of SD

#### Morphological Anomalies

Converging evidence for abnormal increases in the left SMG was noted in two studies. The authors hypothesized that increases in this region reflect “immaturity or altered development” [22] or “reduced synaptic pruning” [18]. In addition, this similar finding points to possible commonalities between the aetiology of speech sound disorders of articulation and phonology and CAS, despite these conditions being traditionally viewed as distinct clinical diagnoses. There may also have been overlap of symptoms between participants from these two studies. In adult neuroanatomical models, the SMG is assumed to play a crucial role in auditory motor and sensorimotor [30] integration. This is a critical region in the somatosensory feedback loop in both the DIVA [31] and HSFC [32] computational models of speech production. Further, a recent repetitive TMS study highlighted the importance of this inferior parietal region and its connections to frontal and motor output areas, in learning and adapting sensorimotor patterns for speech [33]. Structural anomalies in the left SMG are therefore consistent with the hypothesis that SDs arise from abnormal somatosensory feedback or dysfunctional integration between sensory and auditory motor systems.

Morphological anomalies in the STG, a region traditionally involved in auditory processing [30, 34] were reported bilaterally in children with SD [24], but not in the CAS study [22]. In adult models [31, 32], these superior temporal regions are part of the auditory feedback control subsystem. Preston and colleagues argue that children with SD may therefore suffer from abnormal auditory perceptual networks. The observed correlation between speech sound production accuracy and STG volumes in the whole sample (but not the SD subgroup) was seen to support this hypothesis. Yet no data were available on participants’ speech processing performance.

Altogether, the limited structural imaging findings on children with SD converge toward a tendency for atypical increases of grey matter in regions crucial to the system of feedback control during speech production. If confirmed in larger future studies, these findings may indicate that SDs, other than CAS, are associated with both auditory and somatosensory feedback, whereas CAS occurs mainly due to somatosensory feedback deficits. This conclusion remains speculative given that the two groups studied here differed on age and seemingly severity, which may also account for these differences.

In CAS [22], the lack of evidence for morphological anomalies within the typical planning regions (e.g., Broca’s area, insula, ventral premotor cortex) contrasts with both findings on adults with apraxia of speech after stroke [35, 36], and models suggesting these regions play a crucial role in storing motor programs [31, 32]. These neuroanatomical differences could indicate that CAS and Apraxia of Speech are distinct, despite sharing some symptomatology (although see [37] for further discussion of developmental and acquired apraxia). Alternatively, one could argue that the regions involved in speech planning/programming early in speech acquisition and in adulthood differ. Finally, the differences seen between adult and child studies with apraxia of speech may reflect differences in compensation strategies, functional, or structural reorganization patterns. To our knowledge, only Terband and colleagues [38••] have attempted to model childhood motor speech disorders and have begun to predict the possible effects of auditory vs. motor processing deficits on speech errors based on assumptions underlying the DIVA model.

### Functional Anomalies

There was little consistency, and even contrasting findings, between the fMRI studies that used nonsense word repetition [21, 23]. The discrepancies could arise from several causes, the most important being the different phenotype. The study by Liegeois et al., focused on individuals with severe and persistent CAS concomitant with dysarthria and oral dyspraxia; while the other examined individuals with a history of moderate–severe articulation/phonological disorder (where only one individual made speech errors at the time of testing) [23]. Therefore, the hyper-activity of the left hemisphere found in the case of a milder phenotype may be explained by efficient compensatory mechanisms. The authors themselves conclude that adults with “speech sound disorders” rely more on dorsal speech regions [23]. Given that little is known on the exact type of speech errors made by participants (e.g., articulation vs. phonological), generalization of findings to other SD populations remains difficult.

Finally, there was also little agreement on functional anomalies within the cortico-striatal circuits, with both hypo-activity and hyper-activity in the putamen and inferior frontal regions across the three fMRI studies reviewed here— again possibly as a result of different speech symptoms (between and within studies), or different fMRI tasks used.

### Neural basis of LD

#### Morphological Anomalies

Discussion of results remains speculative given the heterogeneity in studies reviewed here. Nevertheless, converging evidence of morphological reductions in the STG/superior temporal sulcus (STS) in either hemisphere suggests an important role for intact auditory processing during typical language development. In the Dual Stream model developed by Hickok and Poeppel [39], the STG and STS are at the interface between the dorsal and ventral routes. A significant body of literature has focused on the hypothesis that language disorders may be born from auditory processing deficits or differences [40••]. The auditory system is obviously critical to healthy speech and language processing, but the exact relationship between language impairment and auditory processing is far from clear [40••]. None of the imaging studies reported here measured auditory processing skills using straight behavioral measures or electrophysiological approaches, making it challenging to interpret the relationship between morphological anomalies of the auditory system and SD or LD any further.

Volumetric reductions in the caudate nucleus [19, 29] (but see [25] for an increase) are consistent with previous findings in the affected members of the KE family [15, 41], where negative correlations with non-word repetition [15] have been reported. Another striatal structure, the putamen, was also found to be enlarged in one study [19], as in the affected KE family members [15] (but see [41]), with larger putamen volumes correlating with poorer language performance. Several models do consider the basal ganglia as crucial to language acquisition given its role in procedural learning [42, 43], but little consensus is evident regarding the specificity of the basal ganglia for language related functions (e.g., grammar learning) [44] vs. more general cognitive development [45]. In addition, although cortico-cortical interaction may be crucial to language acquisition (see section “Commonalities between LD and SD” for further discussion on the basal ganglia), whether cortical or subcortical abnormalities are the primary biomarkers of LD remains unknown.

In addition to whole-brain analyses such as VBM, advances in diffusion weighted imaging and tractography methods now allow us to identify tracts important to the typical development of language. The reductions in FA in the SLF [20] for LD children is noteworthy, and points to atypical development of the dorsal stream [39]; possibly consistent with increased volume in the middle temporal white matter in young children with LD using VBM [25]. It is difficult to conclude whether the relationship between the SLF and language outcome is specific in the tractography study [20], as no other tracts were examined and no correlation with other cognitive functions were conducted. This approach is promising however, as it allows examination of language functions at the network level [46••].

### Functional Anomalies

The most consistent findings of reduced brain activity in the left posterior STG points to both functional and morphological anomalies in this region for people with LD. As mentioned above, this finding would be consistent with abnormal auditory processing in people with SD, although the fact that this region is at the interface between ventral and dorsal streams could explain a wide range of language deficits.

One study also reported hypo-activity in the right putamen and right inferior frontal gyrus— a finding similar to that reported in the left hemisphere of affected KE members [47]. In contrast, increased fMRI activation was noted in the right IFG and caudate in another study [27] (but see [24] for increases in the left IFG). As seen for SD, the inconsistency in fMRI results concerning basal ganglia and inferior frontal activity therefore makes it difficult to disentangle findings associated with compensatory vs. deficit-related brain responses in LD.

### Commonalities and Differences Between LD and SD

The discrepancy in study designs and findings across studies allows us to draw only preliminary conclusions that must be considered with caution.

Although activation in the STG was reported to be abnormally increased in the SD focused studies, reductions were reported in the LD literature. These contrasting findings could imply distinct mechanisms of atypical cortical development in the two conditions. One common finding between LD and SD was the limited evidence for structural abnormalities IFG- *pars opercularis* or *triangularis*, alongside an important role for the temporo-parietal junction in SD and LD. However, again discrepant findings were reported across studies examined here, such as increased grey matter volume in the right hemisphere in LD [25] vs. a left increase in CAS [22].

Findings relating to subcortical structures were also inconsistent between LD and SD populations. Although striatal morphological and functional anomalies were reported across a handful of LD studies, the putamen and caudate nucleus were either not examined or not reported as abnormal in the studies that focused on SD, except in the affected KE family members [21]. Paradoxically, given the putative role of striatal structures in motor learning, more evidence is therefore available for subcortical abnormalities in LD than in SD. Drawing parallels with the KE family findings remains difficult, as the affected members have both SD (primarily childhood apraxia of speech) and LD. In 2005, Ullman & Pierpont [42] suggested that SLI is associated with impaired procedural learning. Reaction time experiments seem to indicate that people with LD have poorer procedural learning skills than their peers (see [48] for a meta-analysis), and that grammatical skills correlate strongly with long-term consolidation of learning [49]. The neuroimaging studies reviewed here present inconsistent results regarding basal ganglia abnormalities, with puzzling negative correlations with language performance. We cannot rule out that subcortical structural abnormalities may be linked to atypical language development, but a causal relationship remains difficult to establish.

In summary, perhaps as predicted from the low co-occurrence of SD and LD, at least in middle childhood [50], the recent neuroimaging evidence does not point towards an obvious common causal pathway for these two conditions.

## General Considerations

The diversity of neuroimaging methods is likely to increase our understanding of developmental SDs and LDs and, in the long term, hopefully provide some answers relevant to the pathways leading from genes to brain to symptomatology. Each method has limitations however, e.g., task-based functional imaging findings are heavily dependent on the task used, and regions of hyper-activation remain difficult to interpret. In VBM analyses, a recurrent question is whether to correct for global volumetric differences or not. Finally, our understanding of both SD and LD is hampered by a lack of developmental models relating how speech and language functions are established between early childhood and into adulthood. Neuroimaging studies have suggested for instance that language processing shifts from an inter-hemispheric to an intra-hemispheric network during development [51], and have revealed asynchrony between the development of ventral and dorsal pathways [52•]. Practically, this means that focusing on left hemisphere regions or tracts may be misleading, especially in younger age groups (see [53] for further evidence of developmental changes in language networks).

## Future Directions

Only large scale prospective longitudinal studies of well-defined clinical subtypes will lead to a more informed picture of the neural bases of LD and SD. Given the change in clinical presentation throughout development [54, 55, 56•], discriminant analyses may also be useful. In addition, functional and effective connectivity approaches have not been used in these populations (yet see [57, 58] for examples in Dyslexia research). These approaches may shed some light on possible network property abnormalities in SD and LD.

## Conclusion

Structural anomalies in SD and LD include a combination of atypical progressive (e.g., “pathologically” larger or thicker grey matter structures) and regressive (e.g., FA and volumetric reductions) processes relative to individuals with typical speech development. Unfortunately, the current lack of consistency in approaches, selection criteria, and age bands make it difficult to extract a consistent developmental trajectory for these conditions.

## Electronic supplementary material

Below is the link to the electronic supplementary material.

Supplementary Figure 1 Flow diagram of papers at each stage of inclusion/exclusion. (DOCX 23.1 KB)

## Footnotes

1 Authors were requested to review literature in this field over the past 12 months. Given the scarcity of literature, the authors extended the search to encompass the past 5 years.
