# Supplementary material for: Neural Correlates of Developmental Speech and Language Disorders: Evidence from Neuroimaging
Source: Curr Dev Disord Rep. 2014 Jun 7;1(3):215–27. doi: 10.1007/s40474-014-0019-1 (PMC4104164; doi:10.1007/s40474-014-0019-1)
Supplement: Supplementary file 1 — Flow diagram of papers at each stage of inclusion/exclusion. (DOCX 23.1 KB) [file 40474_2014_19_MOESM1_ESM.docx]

2602 papers identified from database searches.

2573 papers stage 1 exclusion: based on title /duplicates, 33 remaining.

23 papers stage 2 exclusion: based on abstract/reviewing full text

- 5 polymicrogyria
- 13 no standalone LD or SD group
- 3 no MRI method employed
- 1 conference paper
- 1 only clinical observation of MRI (5/7 with brain lesions)

10 remaining.

4 papers included from manual search, 2606 remaining.

Total of 10 papers included in review

5 involved SD participants

5 involved LD participants
